# The malaria blood stage antigen *Pf*CyRPA formulated with the TLR-4 agonist adjuvant GLA-SE elicits parasite growth inhibitory antibodies in experimental animals

**DOI:** 10.1186/s12936-023-04638-8

**Published:** 2023-07-15

**Authors:** Marco Tamborrini, Anja Schäfer, Julia Hauser, Linghui Zou, Daniel H. Paris, Gerd Pluschke

**Affiliations:** 1 Swiss Tropical and Public Health Institute, Kreuzstrasse 2, 4123 Allschwil, Switzerland; 2 University of Basel, Petersplatz 1, 4001 Basel, Switzerland

## Abstract

**Background:**

*Plasmodium falciparum* cysteine-rich protective antigen (*Pf*CyRPA) is an invasion complex protein essential for erythrocyte invasion. In contrast to several previously clinically tested merozoite vaccine candidate antigens, *Pf*CyRPA is not polymorphic, making it a promising candidate antigen for blood stage vaccine development.

**Methods:**

Mice and rabbits were immunized with vaccine formulations of recombinantly expressed *Pf*CyRPA adjuvanted either with the glucopyranosyl lipid A (GLA) containing adjuvants GLA-LSQ, GLA-SE, GLA-Alum or with Nanoalum. ELISA and indirect immunofluorescence assays (IFA) were used to analyse elicited IgG titers and the *P. falciparum* growth inhibitory activity was determined with a standardized in vitro [^3^H]-hypoxanthine incorporation assay.

**Results:**

In the mouse experiments, the GLA adjuvanted formulations were superior to the Nanoalum formulation with respect to antibody titer development, IFA sero-conversion rates and in vitro parasite growth-inhibitory activity. In rabbits, the highest titers of parasite growth inhibitory antibodies were obtained with the GLA-SE formulation. Comparable mean ELISA IgG endpoint titers were reached in rabbits after three immunizations with GLA-SE adjuvanted *Pf*CyRPA doses of 5, 25 and 100 µg, but with 100 µg of antigen, only two immunizations were required to reach this titer.

**Conclusion:**

*Pf*CyRPA formulated with the human-compatible adjuvant GLA-SE represents an attractive vaccine candidate for early clinical testing in a controlled *P. falciparum* blood stage challenge trial.

**Supplementary Information:**

The online version contains supplementary material available at 10.1186/s12936-023-04638-8.

## Background

An effective vaccine against malaria represents a global health priority and in the past decades, a broad range of malaria vaccine candidates have been clinically tested, including whole inactivated parasites and subunit vaccines against sporozoites, merozoites, and sexual stages of the parasite life cycle [1–3]. Despite limited efficacy [4], the World Health Organization (WHO) has recommended in 2021 use of the pre-erythrocytic vaccine RTS,S/AS01 in children in sub-Saharan Africa and areas with moderate-to-high *Plasmodium falciparum* transmission [5]. The RTS,S vaccine consists of a single fusion protein combining the central repeat region and T-cell epitopes of the *P. falciparum* circumsporozoite protein (*Pf*CSP) with the hepatitis B surface antigen (HbsAg). The fusion protein and free HbsAg assemble spontaneously in ‘RTS,S’ particles, which are formulated with the AS01 adjuvant system.

As the major pathology of malaria is associated with parasite replication within erythrocytes, a merozoite protein that elicits invasion-blocking antibodies would represent an obvious complement to the sporozoite antigen *Pf*CSP. The relatively limited vaccine efficacy of the highly immunogenic merozoite surface proteins initially identified and evaluated as vaccine components may be related to their extensive allelic polymorphism. Furthermore, redundancy in the erythrocyte invasion pathways is also contributing to limit strain-transcending neutralization [6].

In recent years, genomic, transcriptomic, and proteomic data have become available, which have helped to identify the cysteine-rich protective antigen (*Pf*CyRPA) as promising vaccine component among predicted open reading frames expressed at late schizont stage [7]. Together with *Pf*Rh5 and the *P. falciparum* Rh5 interacting protein (*Pf*Ripr), *Pf*CyRPA is part of a ternary invasion complex (RCR complex), which is indispensable for human erythrocyte invasion [8, 9]. Recently, it has been shown that a heterodimeric complex consisting of *P. falciparum* thrombospondin-related apical merozoite protein (*Pf*PTRAMP) and *P. falciparum* cysteine-rich small secreted protein (*Pf*CSS) binds to the RCR complex [10], forming the pentameric PCRCR complex. It is proposed that the transmembrane domain of *Pf*PTRAMP anchors the PCRCR complex to the parasite membrane and that *Pf*CSS mediates interaction of *Pf*PTRAMP with *Pf*Rh5.

*Pf*CyRPA is highly conserved and shows limited immunogenicity in the natural context, as *Pf*CyRPA-specific IgG titers induced during acute *P. falciparum* malaria are decaying rapidly after treatment [11, 12]. However, if *P*fCyRPA is delivered in an appropriate formulation, it is capable of inducing strain-transcendent neutralizing antibodies [13, 14]. Recently, a scalable cost-effective *Pf*CyRPA production process in insect cells has become available, which could be the basis for vaccine production [15]. As subunit vaccines based on recombinant proteins typically require co-administration with an adjuvant, we have evaluated here the use of several human-compatible adjuvants for *Pf*CyRPA vaccine development. In particular, vaccine formulations of *Pf*CyRPA with the TLR-4 agonist GLA-SE were able to elicit high titers of neutralizing antibodies capable of blocking erythrocyte invasion.

## Methods

### Antigen and adjuvants

The recombinant *Pf*CyRPA antigen, a histidine-tagged protein comprising residues 29-362 with N-glycosylation sites being removed, was produced in mammalian HEK 293 cells and purified as previously described [13]. Adjuvants (Table 1) were purchased as single use vials form the Infectious Disease Research Institute (IDRI), now called Access to Advanced Health Institute (AAHI; USA). The adjuvant formulations were provided as 2× or 5× concentrate and mixed with antigen and saline immediately prior to immunization.

**Table 1:** Used adjuvant formulations

Adjuvant	Composition
GLA-LSQ	Liposomes with dioleoylphosphatidylcholine, synthetic TLR-4 agonist glucopyranosyl lipid adjuvant (GLA) and saponin (QS-21)
GLA-SE	Oil-in-water emulsion with squalene, TLR-4 agonist (GLA) and dimyristoyl phosphatidylcholine
GLA-Alum	Aluminum hydroxide with TLR-4 agonist (GLA) and dipalmitoyl phosphatidylcholine
Nanoalum	Aluminum hydroxide nanoparticles with poly(acrylic) acid

### Immunogenicity studies in laboratory mice and rabbits

Mice experiments were carried out in accordance with the national regulations for the protection of animal rights. The protocols were ethically approved by the veterinary office of the county of Basel-City, Switzerland (Permit Number 2375). Groups of 7 week-old specific pathogen-free Crl:NMRI(Han) mice (n = 6) were purchased from Charles River Laboratories (Germany) and used for immunization studies. Mice were immunized three times subcutaneously with 20 µg of recombinant *Pf*CyRPA either without adjuvant or formulated with the adjuvants GLA-LSQ, GLA-SE, GLA-Alum or Nanoalum in intervals of 3 weeks (day 0, 21, and 42). Blood was collected before each immunization and 13 days after the final injection.

New Zealand rabbits were kept, immunized and bled at Kaneka Eurogentec S.A. (Belgium). Groups of rabbits (n = 2) were immunized three times with 40 µg of recombinant *Pf*CyRPA without adjuvant or formulated with the adjuvants GLA-LSQ, GLA-SE or GLA-Alum. Intramuscular immunizations with 0.5 mL injection volume per animal were administered on study days 0, 28 and 56. In dose‑response analysis, groups of rabbits (n = 3) were immunized three times with GLA-SE-adjuvanted *Pf*CyRPA at three different antigen doses (5, 25 and 100 µg *Pf*CyRPA). Blood was collected before each immunization and 4 weeks after the final injection. Total IgG was purified from mouse and rabbit sera using protein A columns (GE Healthcare).

### Enzyme-linked immunosorbent assay (ELISA)

For the measurement of *Pf*CyRPA-specific IgG antibody responses, MaxiSorp™ flat-bottom 96-well ELISA plates (Nunc) were coated with 5 µg/mL of purified recombinant *Pf*CyRPA protein overnight at 4 °C. After blocking and washing, plates were incubated with serial dilutions of sera or purified IgG for one hour at room temperature. The plates were then washed and incubated with goat anti-mouse (Sigma) or anti-rabbit IgG (Jackson ImmunoResearch) conjugated to horseradish peroxidase (HRP) secondary antibodies for 1 h at room temperature. Tetramethylbenzidine was used as substrate (KPL). The reaction was stopped after appropriate time with 0.5 M H_2_SO_4_ and the absorbance was read at 450 nm with the Sunrise absorbance plate reader (Tecan). Data were processed and analysed using GraphPad Prism 8. Humoral immune responses elicited in animals were compared using a one-way ANOVA with Tukey’s test for multiple comparisons.

The distribution of anti-*Pf*CyRPA IgG subclasses was determined by ELISA with alkaline phosphatase-conjugated goat anti-mouse IgG1, IgG2a, IgG2b and IgG3 secondary antibodies (SouthernBiotech).

In avidity ELISA analyses, mouse serum samples were added to *Pf*CyRPA-coated ELISA plates in triplicates at constant dilutions (approx. halfmax titer). After washing, plates were incubated for 15 min with NH_4_SCN diluted in 0.1 M NaH_2_PO_4_ buffer (pH 6) at the following molarities: 5 M, 4 M, 3 M, 2 M, 1 M, 0.5 M, and 0.25 M. After a wash step, the plates were incubated with goat anti-mouse HRP secondary antibodies for detecting antibodies that remained bound to the antigen and processed as described above. The avidity index corresponds to the NH_4_SCN concentration (M) eluting 50% of the bound antibodies.

### Indirect immunofluorescence assay (IFA)

Thin smears of *P. falciparum* (clone 3D7)-infected red blood cells were fixed in 60% methanol and 40% acetone for 2 min at − 20 °C, air-dried and blocked with 3% bovine serum albumin dissolved in phosphate buffered saline. Cells were then incubated with serial dilutions of mouse sera for 1 h at room temperature. After washing, cells were incubated with secondary antibodies specific for mouse IgG conjugated with Alexa Fluor 568 (Invitrogen). Slides were washed, mounted with ProLong™ Gold antifade reagent containing DAPI (Invitrogen) and covered with a coverslip. The immunoreactivity was analysed with a Leica DM-5000B fluorescence microscope using a 60× oil immersion objective lens. Images were processed using Leica Application Suite V4 and Adobe Photoshop 2022.

### In vitro growth inhibition assay (GIA)

The parasite growth-inhibitory activity of purified total serum IgG antibodies was determined using a standardized in vitro [^3^H]-hypoxanthine incorporation assay [16]. *Plasmodium falciparum* 3D7-infected erythrocytes were exposed to increasing concentrations of purified total IgG in culture plates. After 48 h of incubation, 18.5 kBq [^3^H]-hypoxanthine was added to each well. Cultures were incubated for further 24 h before they were harvested onto glass-fiber filters and washed with distilled water. The radioactivity was counted using a Betaplate liquid scintillation counter. The results were recorded as counts per minute per well at each IgG concentration and expressed as percentage of the untreated controls. A four-parameter sigmoidal dose-response curve was fitted to the relationship between log_10_ values of the antibody concentrations and percentage inhibition, and then used to interpolate IC_50_ values. Data were processed and analysed using Microsoft Excel 2016 and GraphPad Prism 8. Statistical analysis was conducted by the ordinary one-way ANOVA with Tukey’s test for multiple comparisons using GraphPad Prism 8 software to calculate p values.

## Results

### Immunogenicity and vaccine efficacy of adjuvanted *Pf*CyRPA vaccine formulations in mice

**Fig. 1:**
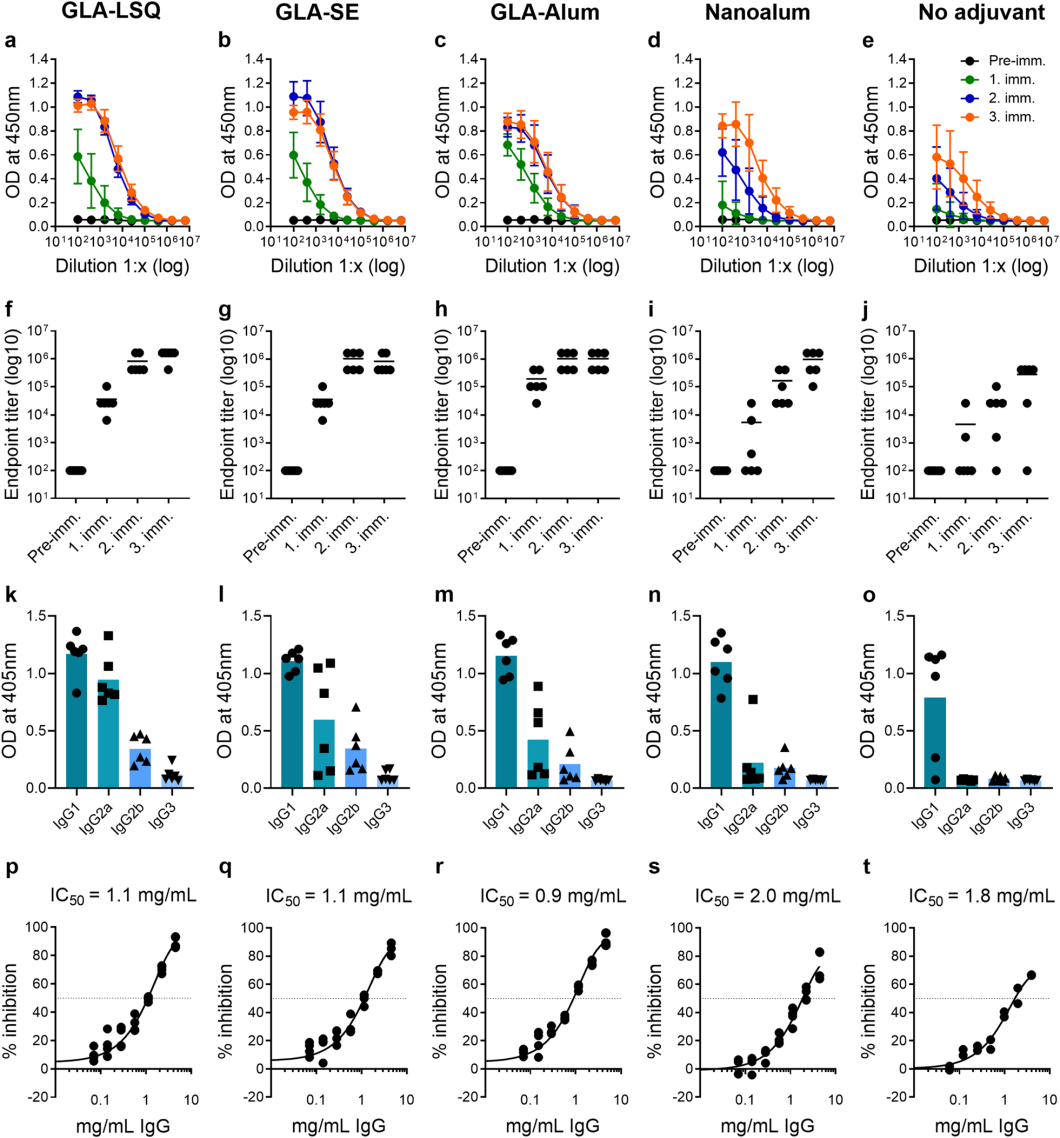
*Pf*CyRPA adjuvanted with either GLA-LSQ, GLA-SE, GLA-Alum or Nanoalum elicited in mice high IgG titers with in vitro parasite growth-inhibitory activity. ELISA readouts (optical density [OD] means ± standard deviations) obtained with serial dilution of mouse sera taken pre-immune and after each immunization are shown in **a**–**e**. Serum anti-*Pf*CyRPA IgG ELISA endpoint titers of individual animals are shown in **f**–**j** and lines represent the mean titer. Determinations of IgG subclass profiles by ELISA using plates coated with recombinant *Pf*CyRPA are shown in **k**–**o**. Sera from mice collected after the third immunization were tested individually at a serum dilution of 1:6400. Shown are OD values obtained with individual sera and means (colored bars). In vitro parasite growth-inhibitory activities of purified total IgG from pooled sera of each group of mice are shown in **p**–**t**. Dots represent duplicate replicates of two independent [^3^H]-hypoxanthine incorporation assays. For each group a four-parameter sigmoidal dose-response curve was fitted to the relationship between the log_10_ of the IgG concentration and percentage inhibition, and then used to interpolate IC_50_ values

Groups of six outbred NMRI mice were immunized three times with 20 µg of recombinant *Pf*CyRPA produced in mammalian HEK 293 cells without adjuvant or formulated with the human-compatible adjuvants GLA-LSQ, GLA-SE, GLA-Alum or Nanoalum, enabling side-by-side comparison of elicited antibody responses (Table 2). Already after the first immunization, all 6/6 mice receiving vaccine formulations adjuvanted with GLA-LSQ, GLA-SE and GLA-Alum had developed anti-*Pf*CyRPA IgG responses with mean ELISA endpoint titers > 35,000 (Fig. 1a–c, f–h; Table 2). While a second immunization led to a substantial titer increase (> 800,000) in these animals, a further increase in IgG titer after the third immunization was observed only in some mice receiving the GLA-LSQ formulation (Table 2). In the Nanoalum group, only 3 out of 6 mice had sero-converted after one immunization and 100% sero-conversion was only observed after the second immunization (Fig. 1d, i). The mean anti-*Pf*CyRPA titer after the second immunization was lower compared to the mean titers of the other adjuvant groups, but increased to a comparable level after the third immunization (Fig. 1i; Table 2). In the unadjuvanted immunization group, the sero-conversion rate was incomplete and titers remained low (< 40,000) after the second immunization. However, the third immunization led to a nearly tenfold titer increase in 5/6 mice. Pre-immune sera showed no reactivity with *Pf*CyRPA in ELISA.

**Table 2:** Humoral immune responses elicited in NMRI mice with adjuvanted formulations of recombinant *Pf*CyRPA

Adjuvant	Dose of adjuvant	Dose of *Pf*CyRPA (µg)	Mean ELISA endpoint titer and ELISA sero-conversion rates						Avidity index	IFA sero-conversion rate	GIA IC_50_ [mg/mL]
			1. imm.		2. imm.		3. imm.		3. imm.	3. imm.	3. imm.
GLA-LSQ	5 µg GLA; 2 µg QS-21	20	35,200	6/6	819,200	6/6	1,433,600	6/6	1.0 ± 0.2	5/6	1.1
GLA-SE	5 µg GLA; 2% squalene	20	35,200	6/6	1,024,000	6/6	819,200	6/6	1.3 ± 0.4	5/6	1.1
GLA-Alum	5 µg GLA; 100 µg Alum	20	192,000	6/6	1,024,000	6/6	1,024,000	6/6	1.4 ± 0.2	5/6	0.9
Nanoalum	100 µg Nanoalum	20	10,800	3/6	166,400	6/6	972,800	6/6	1.1 ± 0.3	1/6	2.0
No adjuvant	–	20	13,600	2/6	36,160	5/6	332,800	5/6	1.1 ± 0.1	3/6	1.8

Mean ELISA IgG endpoint titers of responders and the proportion of animals that seroconverted are shown. The avidity index of the anti-*Pf*CyRPA IgG collected after the third immunization are provided. The avidity index is defined as the NH_4_SCN concentration (M) where 50% of the bound antibodies in ELISA are eluted. Shown are mean values ± standard deviation of six mice per group. The proportion of animals that had developed blood stage parasite cross-reactive IgG after the third vaccination in IFA at a serum dilution of 1:500 is shown. GIA values: The concentrations of purified total IgG from pooled sera giving 50% growth inhibition in GIA (IC_50_) are shown. They were determined in a standardized in vitro [^3^H]-hypoxanthine incorporation assay. Shown are mean values of two independent assays (Fig. 1p–t)

Analyses of the induced *Pf*CyRPA-specific IgG subclass profiles showed a predominance of the IgG1 subclass in all formulations (Fig. 1k–o). To compare the avidity of *Pf*CyRPA specific serum IgG between groups, chaotrope-based avidity measurements were performed with sera collected after the third immunization. No marked differences were found in the mean avidity indices of anti-*Pf*CyRPA IgG responses between the groups (Table 2).

Induction of *P. falciparum* blood stage cross-reactive IgG was analysed by immunofluorescence analysis (IFA) with sera collected after the third immunization. In each of the three groups receiving *Pf*CyRPA adjuvanted with either GLA-LSQ, GLA-SE or GLA-Alum, 84% (5/6) of mice had developed parasite-binding IgG antibodies in IFA (Table 2). Lower IFA sero-conversion rates were observed in the Nanoalum group (1/6) and in the unadjuvanted group (3/6).

13 days after the third immunization, serum was collected by terminal cardiac bleed. IgG was purified from the pooled sera of each group of mice and tested for parasite growth inhibitory activity in an in vitro [^3^H]-hypoxanthine incorporation GIA assay (Fig. 1p–t). Purified IgG from all immunized groups showed substantial dose-dependent parasite in vitro growth-inhibitory activities. IgG preparations of the GLA-LSQ, GLA-SE and GLA-Alum groups were more potent and had lower IC_50_ values (1.1, 1.1 and 0.9 mg/mL of total IgG, respectively) compared to the Nanoalum and unadjuvanted group (2.0 and 1.8 mg/mL of total IgG, respectively), correlating with IFA sero-conversion rates (Table 2).

These analyses of immunological and functional properties of antibodies elicited in mice by differentially adjuvanted *Pf*CyRPA showed that the GLA-LSQ, GLA-SE and GLA-Alum adjuvanted formulations were superior to the Nanoalum formulation with respect to antibody titer development, sero-conversion rates and in vitro parasite growth-inhibitory activity. Further pre-clinical profiling in rabbits thus focused on *Pf*CyRPA formulated with GLA-LSQ, GLA-SE and GLA-Alum.

### Immunogenicity and efficacy of adjuvanted *Pf*CyRPA vaccine formulations in rabbits

**Fig. 2:**
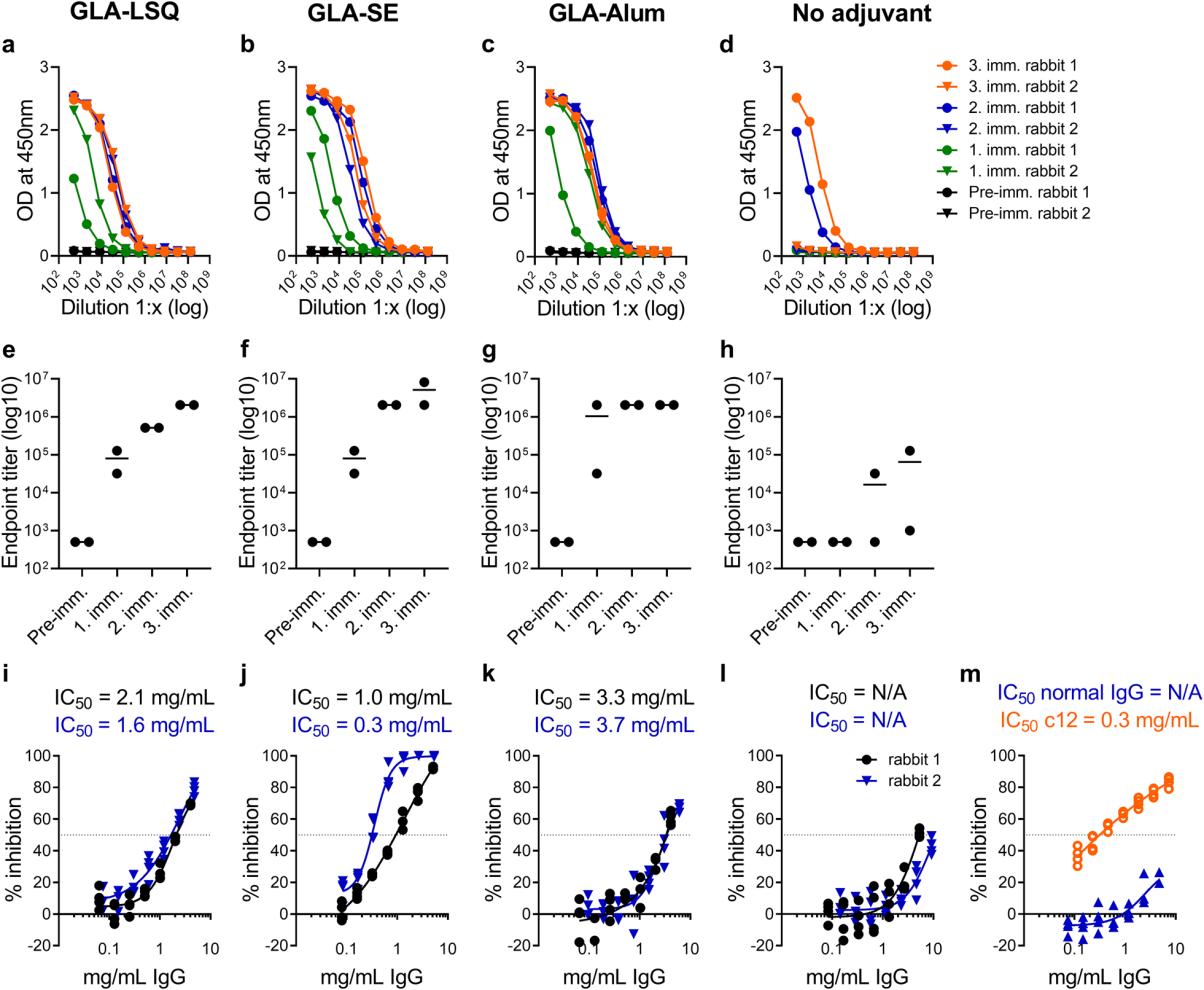
*Pf*CyRPA adjuvanted with either GLA-LSQ, GLA-SE or GLA-Alum elicited in rabbits high IgG titers with in vitro parasite growth-inhibitory activity. ELISA readouts (means ± standard deviations) obtained with serial dilution of sera taken pre-immune and after each immunization are shown in **a**–**d**. Serum anti-*Pf*CyRPA IgG ELISA endpoint titers of individual animals are shown in **e**–**h**, with lines representing the mean titer. In vitro parasite growth-inhibitory activities of purified total IgG from individual rabbit serum samples collected after the final immunization are shown in **i**–**l**. Dots represent duplicate replicates of two independent [^3^H]-hypoxanthine incorporation assays. For each individual rabbit IgG preparation, a four-parameter sigmoidal dose-response curve was fitted to the relationship between log_10_ of the antibody concentration and percentage inhibition, and then used to interpolate IC_50_ values. Purified IgG from non-immunized animals and the parasite inhibitory anti-*Pf*CyRPA mAb c12 were used as controls (**m**). The mean ELISA IgG endpoint titers obtained after the second, but not after the third immunization were significantly higher in the adjuvanted immunization groups when compared to the unadjuvanted group (Additional file 1: Table S1)

Groups of New Zealand rabbits (n = 2 per group) were immunized three times with a dose of 40 µg of recombinant *Pf*CyRPA protein without adjuvant or in combination with GLA-LSQ, GLA-SE and GLA-Alum (Table 3). Sera collected pre-immune and after each immunization were assessed for IgG antibody titers specific for recombinant *Pf*CyRPA by ELISA (Fig. 2a–h; Additional file 1: Table S1). Consistent with the results obtained from the mouse immunization experiment, all animals receiving GLA-LSQ, GLA-SE or GLA-Alum adjuvanted vaccine formulations had developed IgG antibodies that interacted with the *Pf*CyRPA immunogen already after the first immunization. While a second immunization led to a titer increase in all immunized rabbits, booster effects after the third immunization were observed only in some animals receiving the GLA-LSQ and GLA-SE adjuvanted formulations. The IgG endpoint titers obtained after the third immunization were comparable between all adjuvanted groups. In the unadjuvanted immunization group, one animal sero-converted after the second and the other only after the third immunization. Only one rabbit had developed a marked antibody titer after the third immunization, which was much lower than that of the rabbits receiving adjuvanted formulations (128,000 versus > 2,000,000; Table 3). No *Pf*CyRPA-binding IgG was found in the pre-immune sera.

**Table 3:** Humoral immune responses elicited in New Zealand rabbits by adjuvanted formulations of recombinant *Pf*CyRPA

Adjuvant	Dose of adjuvant	Dose of *Pf*CyRPA (µg)	Rabbit #	ELISA serum endpoint titer	Concentration of purified IgG giving half-max binding in ELISA [µg/mL]	GIA IC_50_ [mg/mL]
GLA-LSQ	25 µg GLA; 10 µg QS-21	40	1	2,048,000	0.65	2.1
			2	2,048,000	0.31	1.6
GLA-SE	25 µg GLA; 2% squalene	40	1	8,192,000	0.11	1.0
			2	2,048,000	0.33	0.3
GLA-Alum	25 µg GLA; 500 µg Alum	40	1	2,048,000	0.43	3.3
			2	2,048,000	0.61	3.7
No adjuvant	–	40	1	128,000	1.34	N/A
			2	1000	16.51	N/A

Shown are ELISA IgG endpoint titers of individual serum samples collected after the third immunization and the concentration of purified total IgG that gives half-maximal binding in ELISA. GIA IC_50_ values: The concentrations of purified IgG giving 50% growth inhibition in GIA were determined in a standardized [^3^H]-hypoxanthine incorporation assay. Shown are mean values of two independent assays (Fig. 2i–l)

The parasite growth-inhibitory activity of purified total serum IgG antibodies from individual rabbit serum samples taken after the final immunization was determined using the standardized in vitro [^3^H]-hypoxanthine incorporation assay (Fig. 2i–m; Table 3). Purified IgG from animals that received GLA-SE-adjuvanted *Pf*CyRPA showed substantial dose-dependent parasite in vitro growth-inhibitory activities (Fig. 2j) with IC_50_ values of 0.3 and 1.0 mg/mL of total IgG. Although comparable ELISA IgG titers were induced with the two other tested adjuvanted formulations, the IgG preparations of the GLA-LSQ and GLA-Alum groups were less potent in inhibiting parasite replication and had higher IC_50_ values (2.1, 1.6, 3.3 and 3.7 mg/mL of total IgG, respectively). Purified IgG from the unadjuvanted immunization group showed only low growth-inhibitory activities yielding no IC_50_ values at the tested IgG concentrations. Statistical comparisons of the mean parasite growth-inhibitory activities at an IgG concentration of 2.5 mg/mL indicated significantly higher activities of the GLA-SE group when compared to the GLA-Alum and unadjuvated groups, but not when compared with the GLA-LSQ immunization group (Additional file 1: Table S1 and Fig. S1). No significant growth-inhibitory activities against the parasites were obtained with control IgG from non-immunized rabbits. The parasite inhibitory activity of the anti-*Pf*CyRPA mAb c12 [17] was used as positive control.

### Dose–response analysis in rabbits

**Fig. 3:**
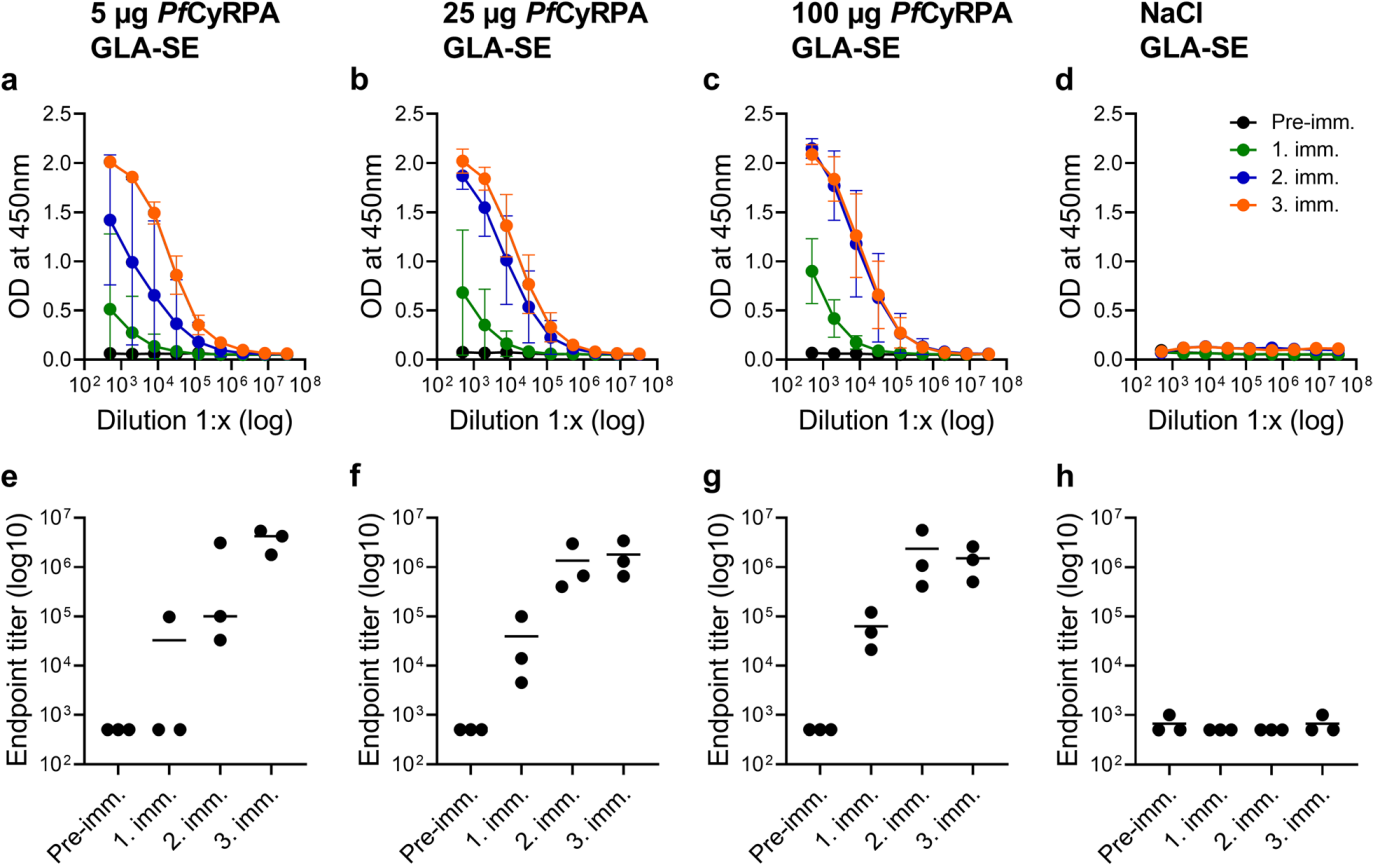
Dose‑response analysis in rabbits. Groups of rabbits (n = 3) were immunized three times with three different doses of *Pf*CyRPA (5, 25 and 100 µg) adjuvanted with GLA-SE. Control animals were immunized with GLA-SE alone. ELISA readouts (means ± standard deviations) obtained with serial dilutions of sera taken pre-immune and after each immunization are shown in **a**–**d**. Serum anti-*Pf*CyRPA IgG ELISA endpoint titers of individual animals are shown in **e**–**h** and lines represent the mean titer

To investigate the effect of different doses of recombinant *Pf*CyRPA on the magnitude of the elicited IgG responses, rabbits were immunized three times with GLA-SE-adjuvanted *Pf*CyRPA at three different antigen doses (5, 25 and 100 µg *Pf*CyRPA). Blood samples were taken pre-immune and after each immunization and analysed by ELISA against *Pf*CyRPA (Fig. 3). In contrast to the 5 µg group, all rabbits receiving the 25 and 100 µg doses developed an anti-*Pf*CyRPA IgG response already after one immunization. While a second immunization led to a titer increase in all dose groups, booster effects after the third immunization were observed mainly in animals of the 5 and 25 µg dose groups. Comparable mean ELISA IgG endpoint titers were reached after the third immunization between all three dose groups. No *Pf*CyRPA-specific IgG responses were found in pre-immune sera and in control animals immunized with the GLA-SE adjuvant alone.

## Discussion and conclusion

The only currently approved malaria vaccine RTS,S/AS01 (Mosquirix), which is targeting the sporozoite antigen *Pf*CSP, is only moderately efficacious in preventing clinical malaria and does not reach the WHO recommended 75% vaccine efficacy [18]. Such a high efficacy may only be reached by a multi-stage second-generation vaccine. As malaria pathology is associated with the parasite blood stages, many efforts have been made to identify essential blood stage antigen candidates. All three components of the *P. falciparum* invasion complex (*Pf*CyRPA, *Pf*RH5 and *Pf*Ripr) can elicit antibodies that inhibit invasion of *P. falciparum* merozoites [7, 13, 17, 19–24]. Here the immunogenicity of adjuvanted formulations of *Pf*CyRPA and the in vitro parasite growth inhibitory activity of the elicited antibodies in a standard GIA were assessed.

In contrast to the ‘classical’ blood-stage vaccine candidate antigens, *Pf*CyRPA shows very limited genetic diversity and immunogenicity. Reasons for this limited immunogenicity are not clear. They may be related to specific features associated with the *Pf*CyRPA protein sequence or to the short period, during which *Pf*CyRPA is exposed in the context of the invasion complex on the surface of the merozoites. However, when delivered together with a suitable adjuvant as recombinant protein to the immune system, *Pf*CyRPA can induce strong parasite growth-inhibitory antibody responses. In mice, two immunizations with a GLA-containing adjuvant were sufficient to induce high antibody titers. To reach comparable titers, three immunizations with a Nanoalum formulation were required. Nevertheless, IgG preparations of the GLA-containing adjuvant groups showed higher GIA activities compared to the Nanoalum group. In mice receiving the antigen without adjuvant, the antibody titer remained low after the second immunization, but increased about tenfold in response to a third immunization, resulting in substantial GIA activity. In contrast, both antibody titer and GIA activity in rabbits receiving the antigen without adjuvant remained low after the third immunization. The significantly highest GIA activity in rabbits was elicited by a formulation containing the GLA-SE adjuvant, which may thus be suitable for the clinical testing of an adjuvanted *Pf*CyRPA formulation. Use of the GLA-SE adjuvant may be more cost-effective than a virosomal formulation [14]. No marked dose dependence in endpoint titers after three immunizations was observed, when rabbits were immunized with GLA-SE formulations containing 5, 25 or 100 µg of *Pf*CyRPA. However, as sero-conversion in the 5 µg group was slower, these data are suggesting to test antigen doses between 20 and 100 µg in a clinical dose finding study. Immunogenicity data in both mice and rabbits are speaking for a testing of a two-dose regimen. While for the experiments described here, *Pf*CyRPA recombinantly expressed in mammalian (HEK293) cells was used, it has been shown recently, that *Pf*CyRPA produced in High Five insect cells has comparable immunological properties [15]. The host cell system used for production had no effect on the reactivity of monoclonal antibodies specific for different epitopes of *Pf*CyRPA and preparations expressed in insect cells are not inferior in eliciting parasite growth inhibitory antibodies [15]. The scalable *Pf*CyRPA production process in insect cells thus appears to be a suitable basis for vaccine production [15].

So far, *Pf*RH5 is the only component of the invasion complex, which has entered into clinical testing [25–27]. Combining *Pf*CyRPA with *Pf*Ripr or *Pf*RH5 may in particular reduce the probability that parasite immune escape variants emerge. Potency of the immune response against the three proteins depends on dose and delivery system and a combination may result in additive or synergistic effects [17, 22–24, 28–31]. Focusing of immune responses on protective epitopes of the candidate antigens may increase vaccine efficacy further [17, 28, 30, 32]. Taken together our data show that *Pf*CyRPA delivered with the human-compatible adjuvant GLA-SE induces high titers of parasite-inhibitory antibodies. This formulation represents an attractive vaccine candidate for early clinical testing in a controlled *P. falciparum* blood stage challenge trial.

## Supplementary Information

**Additional file 1: Table S1.** Humoral immune responses elicited in rabbits with adjuvanted formulations of recombinant *Pf*CyRPA were compared using a one-way ANOVA with Tukey’s test for multiple comparisons using GraphPad Prism 8 software to calculate p values. ****p < 0.0001, **p < 0.005, *p < 0.05; ns not significant. **Figure S1.** Comparisons of the mean parasite growth-inhibitory activities of purified total IgG from individual rabbit serum samples at IgG concentration of 2.5 mg/mL for each formulation group. Only significant differences between groups are shown. **p < 0.005, *p < 0.05.

## Data Availability

The datasets generated during and/or analysed during the current study are available from the corresponding author on reasonable request.

## Abbreviations

*Pf*: *Plasmodium falciparum*
CyRPA: Cysteine-rich protective antigen
WHO: World Health Organization
*Pf*CSP: *Plasmodium falciparum* circumsporozoite protein
HbsAg: Hepatitis B surface antigen
*Pf*Rh5: *Plasmodium falciparum* reticulocyte-binding homolog 5
*Pf*Ripr: *Plasmodium falciparum* Rh5 interacting protein
RCR complex: *Pf*Rh5, *Pf*CyRPA and *Pf*Ripr complex
IgG: Immunoglobulin G
TLR-4: Toll-like receptor 4
GLA: Glucopyranosyl lipid adjuvant
NMRI: Naval Medical Research Institute
ELISA: Enzyme-linked immunosorbent assay
HRP: Horseradish peroxidase
IFA: Indirect immunofluorescence assay
GIA: Growth inhibition assay
IC_50_: Half maximal inhibitory concentration
