# Supplementary material for: The malaria blood stage antigen PfCyRPA formulated with the TLR-4 agonist adjuvant GLA-SE elicits parasite growth inhibitory antibodies in experimental animals
Source: Malar J. 2023 Jul 15;22:210. doi: 10.1186/s12936-023-04638-8 (PMC10349406; doi:10.1186/s12936-023-04638-8)
Supplement: Supplementary file 1 — Additional file 1: Table S1. Humoral immune responses elicited in rabbits with adjuvanted formulations of recombinant PfCyRPA were compared using a one-way ANOVA with Tukey’s test for multiple comparisons using GraphPad Prism 8 software to calculate p values. ****p < 0.0001, **p < 0.005, *p < 0.05; ns not significant. Figure S1. Comparisons of the mean parasite growth-inhibitory activities of purified total IgG from individual rabbit serum samples at IgG concentration of 2.5 mg/mL for each formulation group. Only significant differences between groups are shown. **p < 0.005, *p < 0.05. [file 12936_2023_4638_MOESM1_ESM.docx]

**Additional file 1**

**The malaria blood stage antigen *Pf*CyRPA formulated with the TLR-4 agonist adjuvant GLA-SE elicits parasite growth inhibitory antibodies in experimental animals**

Marco Tamborrini^1,2^, Anja Schäfer^1,2^, Julia Hauser^1,2^, Linghui Zou^1,2^, Daniel H Paris^1,2^, and Gerd Pluschke^1,2^*

^1^Swiss Tropical and Public Health Institute, Kreuzstrasse 2, 4123 Allschwil, Switzerland

^2^University of Basel, Petersplatz 1, 4001 Basel, Switzerland

*Correspondence: Gerd Pluschke, Swiss Tropical and Public Health Institute, Kreuzstrasse 2, 4123 Allschwil,, Switzerland; Gerd.Pluschke@swisstph.ch

**Table S1** (relates to Figure 2 and Table 3). Humoral immune responses elicited in rabbits with adjuvanted formulations of recombinant *Pf*CyRPA were compared using a one-way ANOVA with Tukey’s test for multiple comparisons using GraphPad Prism 8 software to calculate p values. **** p<0.0001, ** p<0.005, * p<0.05; ns not significant.

| **Vaccine formulation comparisons test** | **ELISA endpoint titer after the 1^st^ immunization** | **ELISA endpoint titer after the 2^nd^ immunization** | **ELISA endpoint titer after the 3^rd^ immunization** | **GIA at 2.5 mg/mL purified IgG after 3^rd^ immunization** |
| --- | --- | --- | --- | --- |
| GLA-LSQ vs. GLA-SE | ns | **** | ns | ns |
| GLA-LSQ vs. GLA-Alum | ns | **** | ns | ns |
| GLA-LSQ vs. No adjuvant | ns | **** | ns | * |
| GLA-SE vs. GLA-Alum | ns | ns | ns | * |
| GLA-SE vs. No adjuvant | ns | **** | ns | ** |
| GLA-Alum vs. No adjuvant | ns | **** | ns | ns |

**Figure S1** (relates to Figure 2 and Supplementary Table 1). Comparisons of the mean parasite growth-inhibitory activities of purified total IgG from individual rabbit serum samples at IgG concentration of 2.5 mg/mL for each formulation group. Only significant differences between groups are shown. ** p<0.005, * p<0.05.
